# Comparison of Single and Multiple Intratracheal Administrations for Pulmonary Toxic Responses of Multi-Walled Carbon Nanotubes in Rats

**DOI:** 10.3390/nano14242019

**Published:** 2024-12-16

**Authors:** Hideki Senoh, Masaaki Suzuki, Hirokazu Kano, Tatsuya Kasai, Shoji Fukushima

**Affiliations:** 1 National Institute of Occupational Safety and Health, Japan Organization of Occupational Health and Safety, Fujisawa 251-8555, Kanagawa, Japan; 2 National Institute of Occupational Safety and Health, Japan Organization of Occupational Health and Safety, Kawasaki 214-8585, Kanagawa, Japan; 3 Former Japan Bioassay Research Center, Hadano 257-0015, Kanagawa, Japan

**Keywords:** nanomaterial, multi-walled carbon nanotube, carbon nanotube, intratracheal administration, rat lung toxicity

## Abstract

The purpose of the present study is to contribute to the establishment of a standard method for evaluating the adverse effects of nanomaterials by intratracheal administration. Low and high doses of multi-walled carbon nanotubes (MWCNTs) were administered to rats in a single administration or the same final dose as the single administration but divided over four administrations. Bronchoalveolar lavage examination on day 14 showed an inflammatory reaction and cytotoxicity in the lung, generally greater at the higher dose, and tending to be greater in the rats with four administrations at both the low and high doses. Histopathologic findings showed increased alveolar macrophages and MWCNT deposition (fibers phagocytosed by alveolar macrophages and fibers that were not phagocytosed) in the alveolar space, granulomatous changes, and MWCNT deposition in bronchus-associated lymphoid tissue (BALT) and lung-related lymph nodes on days 14, 28, and 91. In addition, alveolar type II epithelial hyperplasia was observed on day 91, and fibrosis of the alveolar wall was observed on days 28 and 91. Fewer alveolar macrophages with phagocytosed MWCNTs were present at day 91 compared to day 28. MWCNT deposition tended to be higher in the BALT after a single administration, whereas deposition was higher in the lung-related lymph nodes after four administrations. MWCNTs were considered to be transported from the lungs or BALT to the lymph nodes over time. There were no significant differences in MWCNT deposition in the lung after the single administration compared with four administrations at either the low or high doses, and the histopathological findings were similar after single and four administrations, at both the low and high doses. Based on the above findings, a toxicity evaluation of the nanomaterials can be sufficiently performed by intratracheal administration, even with a single intratracheal administration.

## 1. Introduction

Inhalation toxicity studies of nanomaterials are extremely important for occupational health, because nanomaterials may be inhaled in the workplace and show respiratory effects. Artificial nanomaterials, especially carbon-based nanomaterials, can be produced with various types of structures, surface properties, surface areas, and aspect ratios. Impurities introduced during production can also affect toxicity [1,2,3]. We have previously reported the subacute and subchronic toxicity of multi-walled carbon nanotubes (MWCNTs) by inhalation exposure in rats [1]. It took a great deal of effort, ability, time, and expense to develop an inhalation exposure device, and the cost of conducting the animal inhalation studies was extremely high. Today, many new nanomaterials are being developed. Consequently, it is extremely difficult and impractical to obtain hazard information on many nanomaterials by inhalation studies in animals. Therefore, it is necessary to develop a simple and inexpensive experimental method that can replace inhalation studies. The intratracheal administration of nanomaterials is useful for screening nanomaterials to evaluate their toxicity because it is inexpensive, can be used by numerous research groups, and yields pertinent information on the inhalation toxicity of the tested materials [4,5,6,7,8,9,10,11,12,13,14,15,16]. To be used by different research groups, the standardization of the intratracheal administration method is required. To contribute to the establishment of this standardization, we previously conducted a study to determine whether there is a difference in the biological response in rats between the administration of a single dose of nickel oxide nanoparticles and the same dose divided over four administrations (multiple administrations) [4]. In the present study, we report the results of our study to determine whether there is a difference in the toxicity of MWCNTs administered in single dose or the same dose divided over four administrations using low and high doses of MWCNTs.

## 2. Materials and Methods

### 2.1. Test Substance

A straight type of MWCNT (MWNT-7; Lot No. 071223-43, Hodogaya Chemical Co., Ltd., Tokyo, Japan) was used for this study. According to Hodogaya Chemical, the MWNT-7 fibers were generated using a floating chemical vapor deposition (CVD) process with a carbon purity > 99.6%. The MWCNTs had a nominal average diameter of 73 nm, an aspect ratio greater than 100, and a surface area of 24 m^2^/g. The MWCNT suspensions used for intratracheal administration were prepared by weighing the MWCNTs and adding the fibers to PBS containing 0.1% Tween 80. The solution was sonicated using an ultrasonic homogenizer for 20 min (VP-30S, 20 kHz, 300 W, TAITEC Co., Ltd., Koshigaya, Saitama, Japan) to suspend the MWCNTs. The solution was dispersed again with ultrasonic waves before administration, and the dispersed MWCNTs were administered to the rats within 1 min after ultrasonication. The MWCNT suspensions used in the present study were sampled (before and after sonde permeation) and photographed under a Field-Emission Scanning Electron Microscope (FE-SEM: SU-8000, Hitachi High-Tech Co., Ltd., Tokyo, Japan) to measure their size. The lengths and diameters of 500 MWCNT fibers in the filtered MWCNT suspension were measured using these photographs.

### 2.2. Animals and Husbandry

Male F344/DuCrlCrlj rats (SPF) (Chares River Laboratories Japan, Inc., Atsugi, Japan) were used. The animals, 11-week-old at arrival, were quarantined and acclimated for 1 week. Animals were housed in specific pathogen-free air-conditioned animal rooms maintained at 23 ± 22 °C and 55 ± 15% humidity. The fluorescent lighting was controlled automatically to give a 12 h light/dark cycle. The animals were fed a CRF-1 pellet diet (sterilized by gamma-ray irradiation, Oriental Yeast Co., Ltd., Tokyo, Japan) and given filtered and ultraviolet-irradiated municipal water ad libitum.

The present studies were conducted in compliance with the basic policy on the conduct of animal experiments in institutions under the control of the Japanese Ministry of Health, Labour and Welfare (Notification by Director of Health Sciences Division, Ministers Secretariat, MHLW) and policies on animal experiments (established by the Japan Bioassay Research Center; JBRC). In addition, this study was approved by the Animal Experiment Committee of the JBRC (approved No. 0111 and 0112). The animals were taken care of in accordance with the Guide for Animal Experimentation (Japanese Association for Laboratory Animal Science).

### 2.3. Experimental Design

The dose setting of the present experiment was based on the results of a 13-week inhalation exposure study of MWCNTs in rats conducted at the JBRC [1]. The 13-week whole-body inhalation exposure study of MWCNTs in rats was conducted at exposure concentrations of 0 (control), 0.2, 1, and 5 mg/m^3^ for 6 h per day, 5 days per week. In the present study, the area under the lung concentration–time curve (AUC) was calculated from the lung deposition in the group exposed by inhalation to 1 mg/m^3^. Deposition was assumed to increase at a fixed rate over time. For the low-dose group, we used the 13 week value of the fiber deposition according to the AUC calculations, which was 240 µg/kg bw. The high-dose group was defined as twice the dose of the low-dose group, 480 µg/kg body weight.

**Figure 1:**
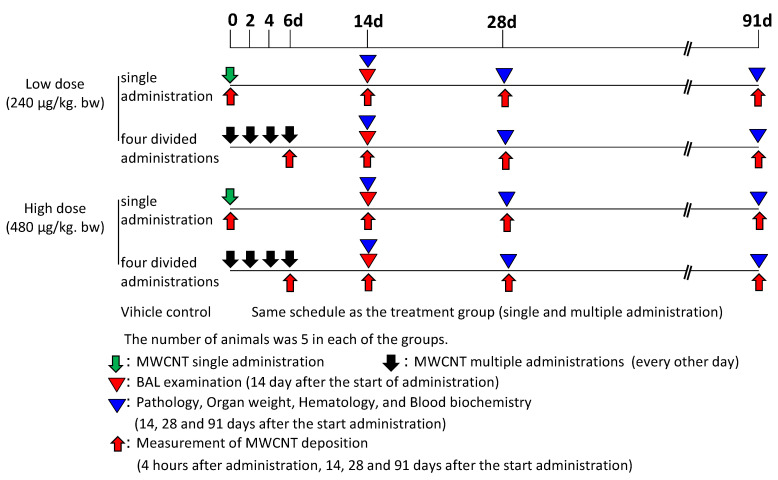
Experimental design.

The study design is presented in Figure 1. The male rats, a total of 205, were 12 weeks old with a mean body weight of 262 g at the commencement of the experiment. The MWCNTs were treated with PBS containing 0.1% Tween 80 as described in “Section 2.1 Test Substance”. The rats were divided into 6 groups: One group of rats received a single administration of MWCNTs at 240 µg/kg bw. A second group of rats received four administrations (multiple administrations) of MWCNTs (60 µg/kg bw per dose: 240 µg/kg bw total). A third group of rats received a single administration of MWCNTs at 480 µg/kg bw. A fourth group of rats received four administrations of MWCNTs (120 µg/kg bw per dose: 480 µg/kg bw total). A fifth group of rats received a single dose of PBS containing 0.1% Tween 80. A sixth group of rats received four doses of PBS containing 0.1% Tween 80. Rats receiving 4 doses of MWNCT or PBS/Tween 80 were administered MWCNTs or PBS/Tween 80 every other day. Table 1 shows the number of animals used for each examination and each date of examination (also see Figure 1). Five rats per group were used for each examination. Blood sampling for hematology and blood biochemistry and pathological examinations were performed on days 14, 28, and 91 after the start of administration. Bronchoalveolar lavage (BAL) examinations were performed on day 14 after the start of administration. Analyses of MWCNT lung burden (measurement of MWCNT deposition) were performed 4 h after the last administration and on days 14, 28, and 91 after the start of administration. For pathology, a BAL and MWCNTs analysis was performed in all lung lobes, and different animals were used because the intratracheal administration did not result in a homogeneous distribution of lung lobes.

### 2.4. Intratracheal Administration Method

MWCNTs were administered intratracheally under isoflurane (2–3%) inhalation anesthesia using a 1 mL disposable syringe and MicroSprayer (IA-1B-R, Penn-Century, Inc., Wyndmoor, PA, USA). The MicroSprayer was held at a 45–60 degree angle with the animal’s head in the up position. The device was orally inserted into the trachea to a depth of approximately 6 cm from the corner of the mouth [13]. The administration volume per animal was 1 mL/kg bw.

### 2.5. Clinical Observations, Body Weights, and Hematological and Blood Biochemical Analyses

The rats were observed daily for clinical signs. Body weights were measured daily in all rats during the administration period, weekly thereafter, and on each necropsy day. Hematological and blood biochemistry analyses and histopathological examinations were performed on days 14, 28, and 91 after the start of administration (Figure 1). The blood samples were analyzed with an automatic cell analyzer (ADVIA 120, Siemens Healthcare Diagnostics Inc., Tarrytown, NY, USA) for hematology, a blood coagulation analyzer (Sysmex CA-510: Sysmex Corporation, Hyogo, Japan) for the blood coagulation test, and a biochemical analyzer (Hitachi 7080, Hitachi, Ltd., Ibaraki, Japan) for blood biochemistry. The hematology included the measurement of red blood cells, hemoglobin, hematocrit, mean corpuscular volume, mean corpuscular hemoglobin, mean corpuscular hemoglobin concentration, platelets, reticulocytes, prothrombin time, activated partial thromboplastin time, white blood cells, and differential leukocytes count [4]. The blood biochemistry included measurements of total protein, albumin, globulin, the albumin/globulin ratio, total bilirubin, glucose, total cholesterol, triglyceride, phospholipid, aspartate transaminase, alanine transaminase, lactate dehydrogenase (LDH), alkaline phosphatase (ALP), γ-glutamyltransferase (γGTP), creatine kinase, urea nitrogen, creatinine, sodium, potassium, chloride, calcium, inorganic phosphorus, and total bile acid [4].

### 2.6. Cytological and Biochemical Analyses of Bronchoalveolar Lavage (BAL)

BAL examinations were performed in all lung lobes and trachea in each group on day 14 after the start of administration (Figure 1). The animals were anesthetized by 2–3% isoflurane (Mylan Inc., Tokyo, Japan) inhalation and euthanized by exsanguination from the abdominal aorta. The BAL was collected using the following procedure [4,13]. A total of 14 mL (7 mL × 2) of saline was injected into the lungs from a height of 30 cm above the animal, and the saline was collected by placing a collection tube lower than the animal and above the animal allowing it to drain spontaneously from the lungs. This procedure was repeated two times. The collected BAL fluid volume was measured. For the cytological examination of the BAL, total cells were counted with an automatic cell analyzer, described above, and smear preparations were made for the determination of cell classification (neutrophil, lymphocyte, alveolar macrophage, eosinophil, and basophil) [4]. For the BAL biochemistry, the BAL was centrifuged and the total protein, albumin, LDH, ALP, and γGTP levels were measured. Analyses of the BAL used the same equipment as the hematological and blood biochemistry analyses [4].

### 2.7. Organ Weights, Macroscopic and Histopathological Examinations

Organ weights of the liver, kidneys, lungs, spleen, and brain were measured (absolute weight and body weight ratio). Macroscopic examinations of all organs and histopathological examinations of the liver, kidneys, lungs, spleen, brain, and lung-related lymph nodes (posterior mediastinal lymph nodes and parathymic lymph nodes) were also performed. For the histopathological examination, tissues were fixed in 10% neutral buffered formalin (lungs were divided into individual lobes) and embedded in paraffin (the lung lobes were embedded to obtain the largest possible area). Tissue sections of 3 µm thickness were prepared from all organs and stained with hematoxylin and eosin (H & E) for histopathological examination.

### 2.8. Lung Deposition (Lung Burden) Analysis

MWCNT deposition in the lungs and trachea was measured 4 h after the last administration and 14, 28, and 91 days after the start of administration (Figure 1). The lungs and trachea were removed, and the lungs were cut into 5 lobes. Each was weighed and stored in 10% neutral buffered formalin. MWCNT deposition in the samples was analyzed by using hybrid markers, developed by Ohnishi et al. [17]. The method uses the digestion of lung tissues with a strong alkali solution and marks MWCNTs with benzo[*ghi*]perylene.

## 3. Results

### 3.1. Properties of MWCNTs

**Figure 2:**
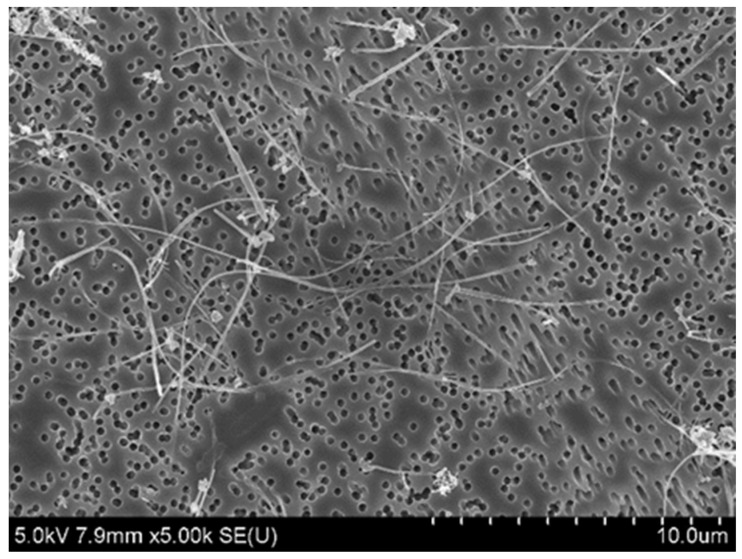
SEM image of MWCNTs in suspension. In suspension (480 µg/mL), MWCNTs were well dispersed on the filtered samples of the suspension.

Figure 2 shows an SEM image of the MWCNTs used in the present study. The SEM image shows 480 µg/mL MWCNTs suspended in PBS containing 0.1% Tween 80. The MWCNTs were well dispersed. The average fiber length (geometric mean ± geometric standard deviation) was 5.4 ± 1.8 µm and the fiber diameter was 75.8 ± 1.4 nm before passing through the spray sonde, and the average fiber length and diameter were 5.1 ± 1.8 µm and 76.4 ± 1.4 nm after passing through the spray sonde. In the 480 µg/mL MWCNT suspension, there was no difference in these fiber parameters before and after passage through the spray sonde.

### 3.2. Clinical Observations and Body Weights

**Figure 3:**
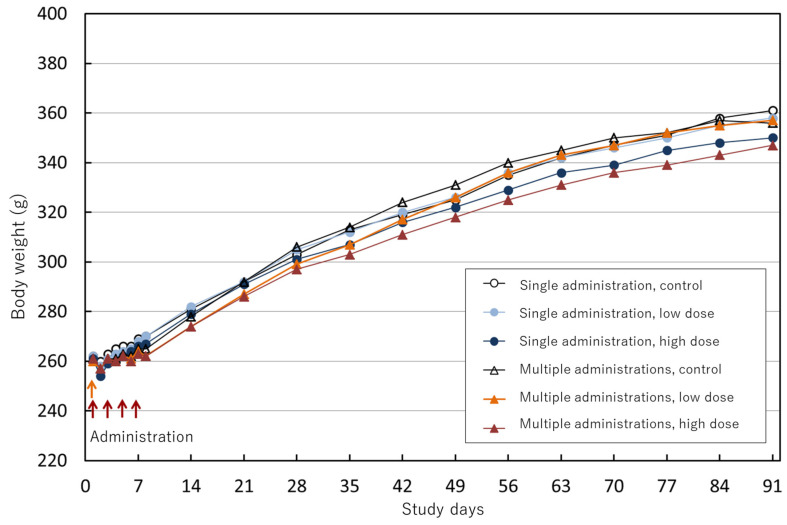
Body weight curves of rats for 91 days.

No abnormal clinical findings were observed in any of the groups throughout the observation period. Both the vehicle control and the treatment groups showed a very slight decrease in body weight on the day following administration, but this was a temporary change, and body weight increased steadily after the end of administration. There was a trend toward the suppression of weight gain in the high-dose groups for both the single- and four-administrations groups, but there were no significant differences between any of the groups (Figure 3).

### 3.3. Cytological and Biochemical Analyses of the Bronchoalveolar Lavage

**Figure 4:**
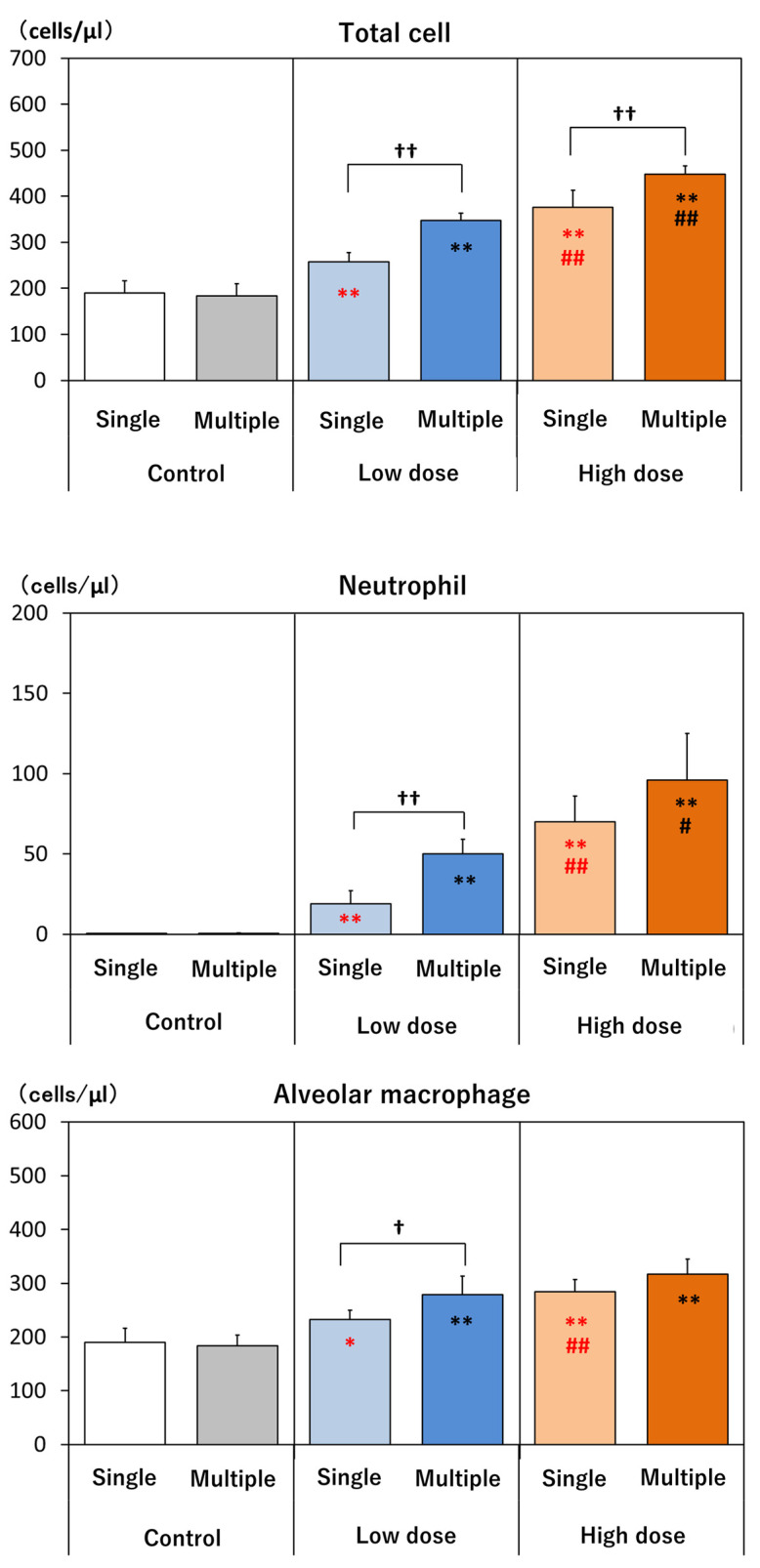

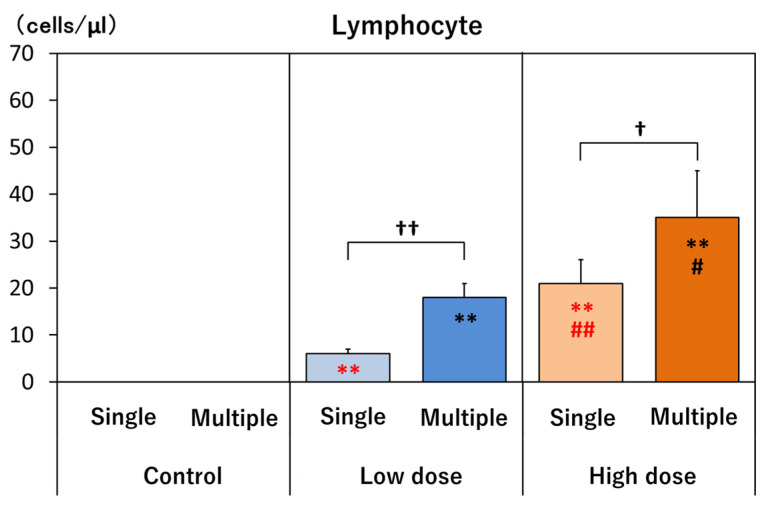
Cytological BAL analysis. Total cells, neutrophils, alveolar macrophages, and lymphocytes at day 14. *: *p* < 0.05, **: *p* < 0.01, significantly different as compared to respective controls (vehicle control), Dunnett test. #: *p* < 0.05, ##: *p* < 0.01, significantly different as compared to low-dose group, *t*-test. †: *p* < 0.05, ††: *p* < 0.01, significantly different in multiple-administration test as compared to single administration, *t*-test.

On day 14, BAL fluid was collected from the lungs. The collected BAL fluid volume (see Section 2.6) did not differ between the control and the treated groups. Total cell count, neutrophil, alveolar macrophage, and lymphocyte counts were increased in all treatment groups compared to the control, and except for alveolar macrophages in the high-dose multiple administration group, these parameters were significantly increased in the high-dose groups compared to the low-dose groups: there was an increase in alveolar macrophages in the high-dose multiple administration group compared to the low-dose multiple administration group, although this increase was not statistically significant. In addition, a comparison of the single- and the four-administration groups showed an increase or increasing trend in the four-administrations groups (Figure 4). Eosinophils and basophils were rarely observed or not seen in the control and dosed groups.

**Figure 5:**
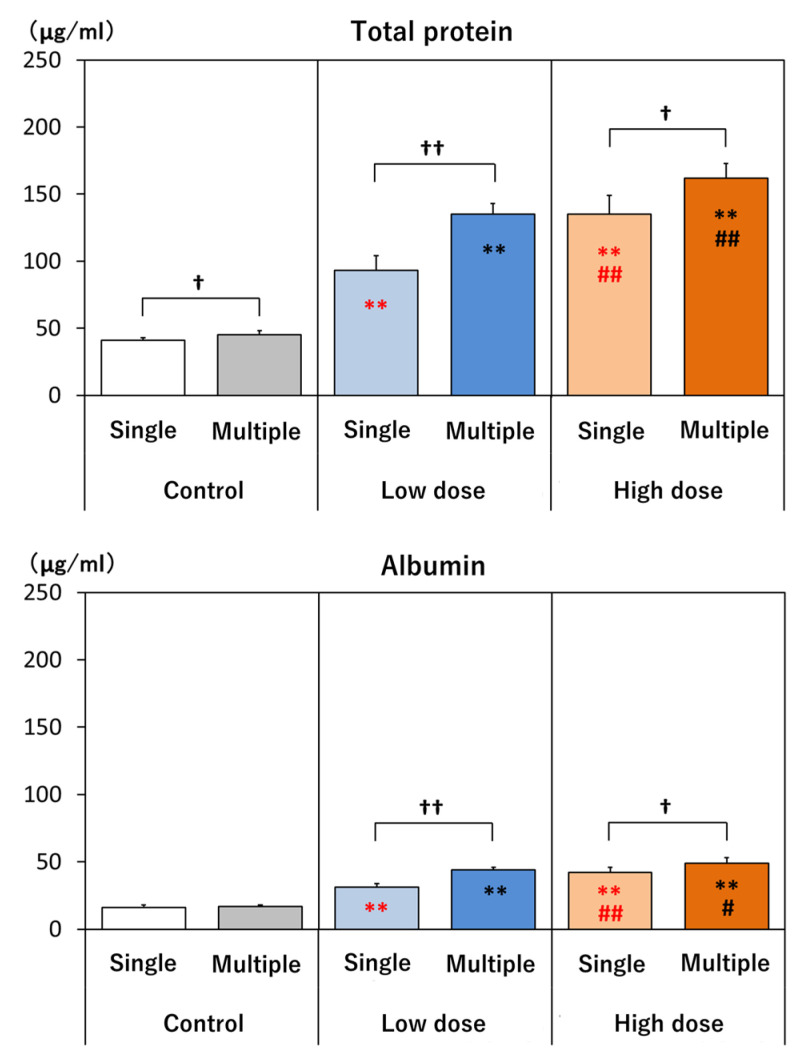

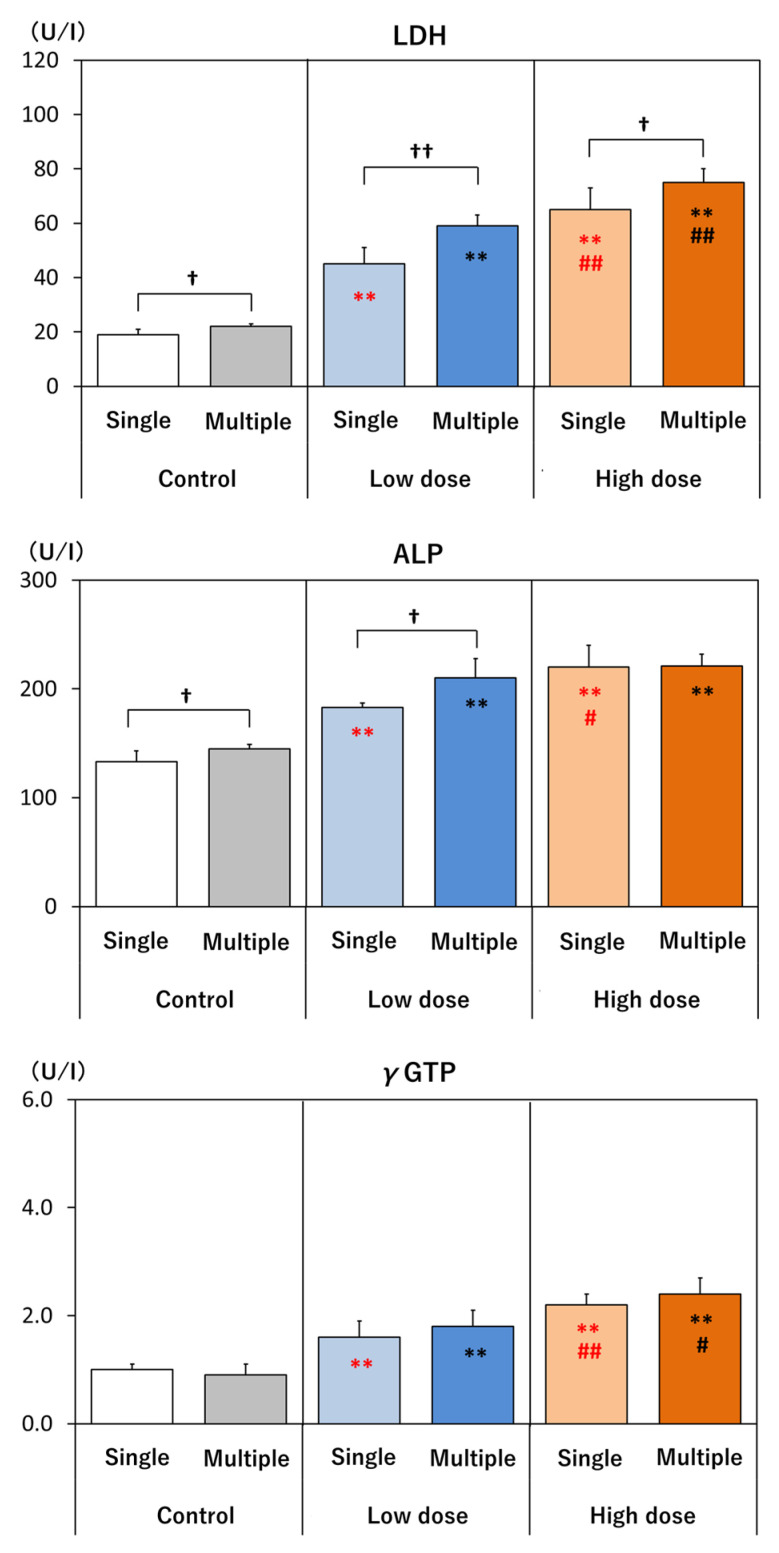
Biochemical BAL analysis. Total protein, albumin, LDH, ALP, and γGTP at day 14. **: *p* < 0.01, significantly different as compared to respective controls (vehicle control), Dunnett test. #: *p* < 0.05, ##: *p* < 0.01, significantly different as compared to low-dose group, *t*-test. †: *p* < 0.05, ††: *p* < 0.01, significantly different in multiple-administration test as compared to single administration, *t*-test.

A biochemical examination of the BAL on day 14 showed that total protein, albumin, and LDH were elevated in all treatment groups and were higher in the high-dose groups than in the low-dose groups (Figure 5). Total protein, albumin, and LDH were also higher in the four-administrations groups compared to the single-administration groups. ALP was elevated in all treatment groups. ALP was higher in the high-dose than in the low-dose group after a single administration of MWCNTs. However, there was no difference between the high-dose four-administration group and the low-dose four-administration group (Figure 5). ALP was also higher in the low-dose four-administrations group compared to the single-administration group, but not in the high-dose four-administrations group compared to the single-administration group. γGTP was also elevated in all treatment groups. In both the single-administration and four-administration groups, γGTP was higher in the high-dose groups than in the low-dose groups. However, in both the low-dose groups and the high-dose groups, γGTP was not higher in the four-administration groups compared to the single-administration groups (Figure 5).

### 3.4. Hematology and Blood Biochemistry

The hematology showed no differences in any of the treatment groups compared to their respective control groups. In addition, although the analysis of blood biochemistry showed significant differences, there were no toxicologically significant changes.

### 3.5. Macroscopic Findings and Organ Weights

**Figure 6:**
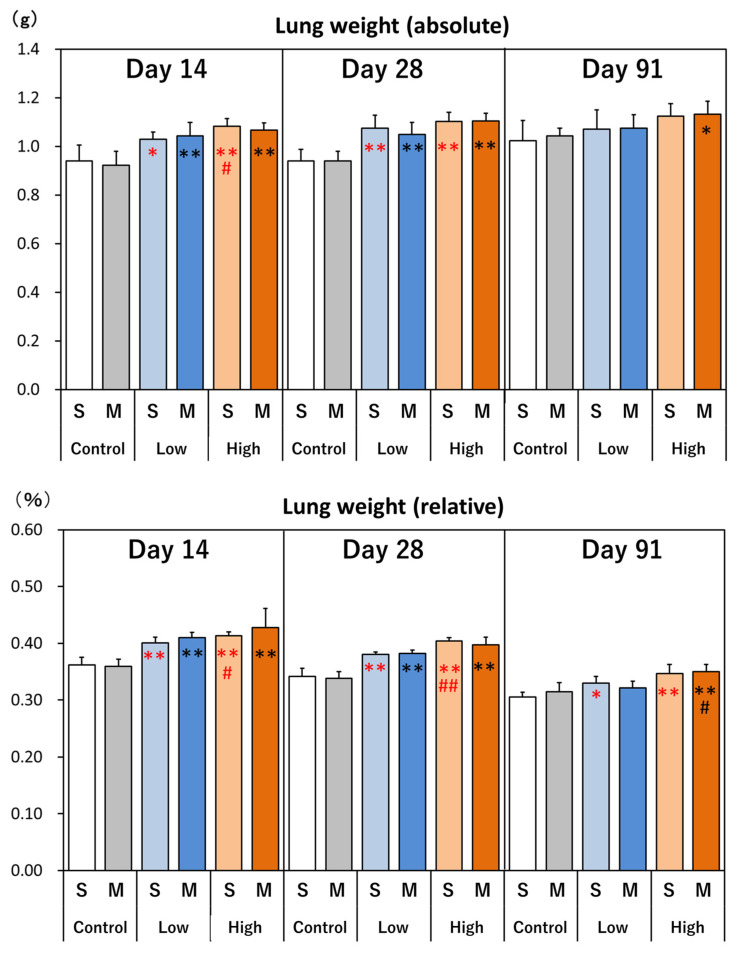
Lung weight. Absolute lung weight, relative lung weight. *: *p* < 0.05, **: *p* < 0.01, significantly different as compared to respective controls (vehicle control), Dunnett test. #: *p* < 0.05, ##: *p* < 0.01, significantly different as compared to low-dose group, *t*-test. 1: Single administration. 4: Four divided administrations (multiple administration).

The macroscopic findings revealed black spots/zones on the lung surfaces of all the MWCNT-treated groups on days 14 and 28, but on day 91 black spots/zones were not observed on the lung surfaces. Lung weights (absolute and relative weight) increased in all dosed groups on days 14 and 28, and on day 91 there was a slight trend towards an increase in the high-dose groups. There were no differences between the single-administration and the four-administrations groups (Figure 6). Except for the lungs, there were no macroscopic findings in other organs and no changes in organ weights.

### 3.6. Histopathological Findings

**Figure 7:**
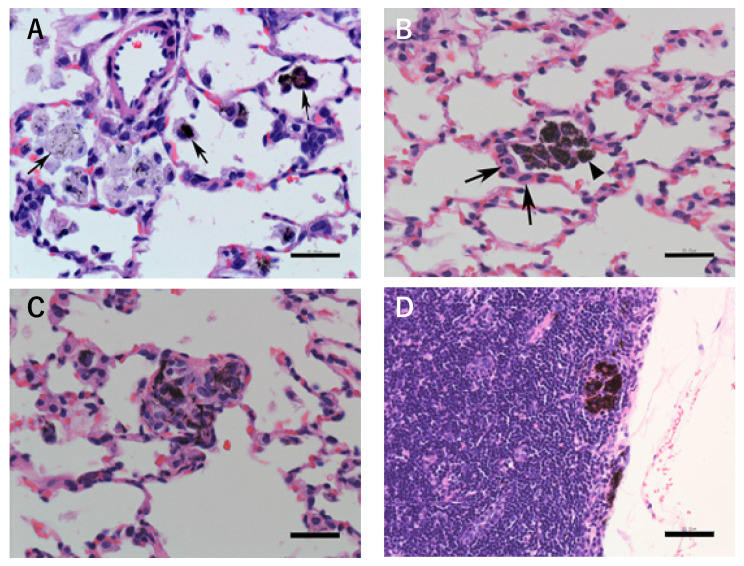
Histopathologic finding. (**A**) Lung: increase in alveolar macrophages (arrow: MWCNTs phagocytosed by macrophages). Macrophages with phagocytosed MWCNTs (dispersed or agglomerated). Four administrations, high dose (total 480 µg/kg), day 14, H&E stain, Bar: 30 µm. (**B**) Lung: alveolar type II epithelial hyperplasia (arrow: alveolar type II epithelial cells, arrowhead: MWCNTs phagocytosed by macrophages). Macrophages with phagocytosed MWCNTs (large amounts) and surrounded by alveolar type II epithelial cells. Four administrations, high dose (total 480 µg/kg), day 91, H&E stain, Bar: 30 µm. (**C**) Lung: granulomatous change involved with aggregated MWCNTs. Four administrations, high dose (total 480 µg/kg), day 91, H&E stain, Bar: 30 µm. (**D**) Lymph node (lung-related lymph node): deposition of MWCNTs and involvement of aggregated MWCNTs. Four administrations, high dose (total 480 µg/kg), day 91, H&E stain, Bar: 30 µm.

In the MWCNT-dosed groups, histopathological changes were observed in the lungs and lung-related lymph nodes (Table 2 and Figure 7). In the lungs, alveolar macrophages (phagocytosed MWCNTs) were increased in all of the rats of all the dosed groups (both low and high doses, single and four administrations) on days 14, 28, and 91. The grade was moderate at the high-dose levels and slight at the low-dose levels on days 14 and 28, and slight at both the low- and high-dose levels on day 91. Deposition of MWCNT fibers (phagocytosed by alveolar macrophages and MWCNT fibers that were not phagocytosed) in the alveolar space was seen in all rats in all dosed groups, and the grade was slight in all cases. MWCNT fibers tended to be found less frequently in the right anterior lobe and anterior side of the left lung, and more frequently in the right posterior and accessory lobes and posterior side of the left lung. Thus, the distribution of MWCNTs in each lung lobe was not well dispersed. This was true for both the single-administration groups and the four-administrations groups. Deposition of MWCNTs in bronchus-associated lymphoid tissue (BALT) was present only in the high-dose single-administration rats on day 14. However, on days 28 and 91, deposition of MWCNTs in the BALT was observed in the low- and high-dose single- and four-administration rats. The grade was slight in all cases. Alveolar type II epithelial hyperplasia was present in all low- and high-dose groups on day 91, and the grade was slight in all cases. Granulomatous changes in the lungs were seen on day 91 for the low-dose single-administration group, and on days 14, 28, and 91 for the high-dose single- and four-administrations groups. All rats in both the high-dose single-administration and the high-dose four-administration groups had granulomatous changes in the lungs on day 91. The grade was slight in all cases. Fibrosis of the alveolar wall was observed in a small number of rats in the high-dose groups on days 28 and 91, and the grade was slight in all cases. In the lung-related lymph nodes (posterior mediastinal lymph node and parathymic lymph node), deposition of MWCNTs was observed only in the high-dose four-administrations groups on days 14 and 28. On day 91, the deposition of MWCNTs in lung-related lymph nodes was seen in the low-dose four-administrations group and the high-dose single- and four-administrations groups. The grade was slight in all cases.

Thus, the histopathological findings in the lungs showed almost no differences in the single- and four-administrations groups. In contrast, the deposition of MWCNTs in the BALT was elevated in the single-administration groups, and deposition in lung-related lymph nodes was elevated in the four-administrations groups. This trend was seen in the high-dose groups on days 14 and 28 and in the low-dose groups on day 91. The histopathological results did not show any dose-related changes except in the lungs and lung-related lymph nodes.

### 3.7. MWCNT Deposition in the Lung (Lung Burden)

**Figure 8:**
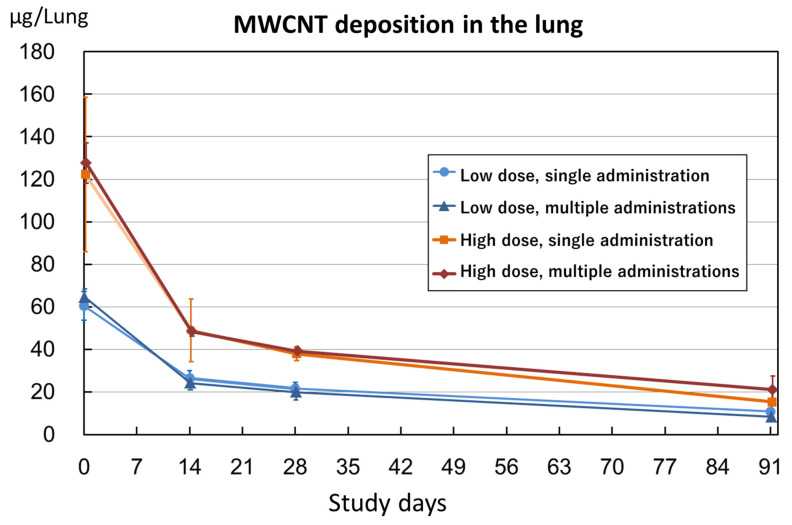
MWCNT deposition in the lung.

The trend of the decreasing deposition of MWCNTs in the lung is shown in Figure 8. The MWCNT fiber deposition at 4 h after the start of administration was consistent with the average total dose per animal (low dose: 63 µg single administration, 63 µg four administrations; high dose: 125 µg single administration, 126 µg four administrations). The amount of MWCNT deposition in the lungs decreased sharply on day 14 compared to 4 h after administration, and this was then followed by a gradual decline. Deposition decreased linearly from day 14 to days 28 and 91. There was no significant difference in the amount of MWCNT deposition and its removal from the lung between the single- and four-administration groups in either the low- or high-dose groups.

**Figure 9:**
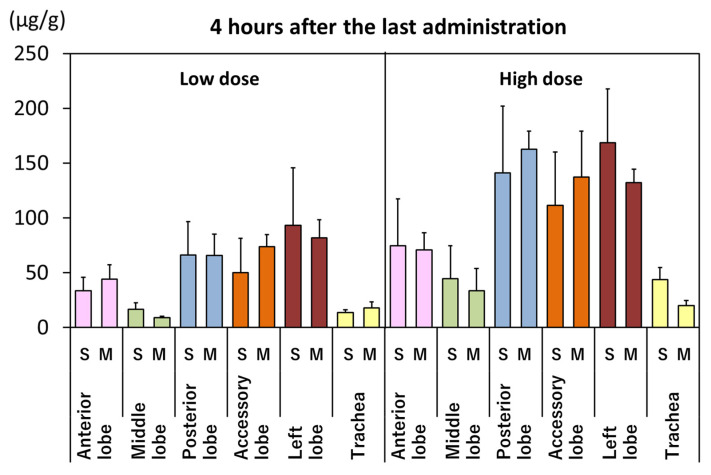

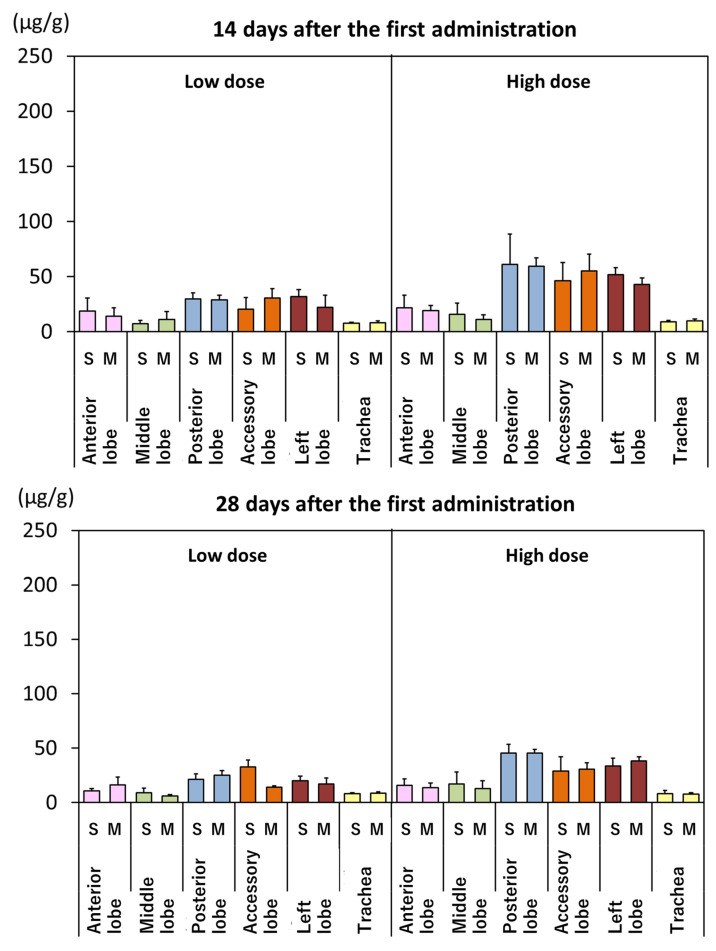

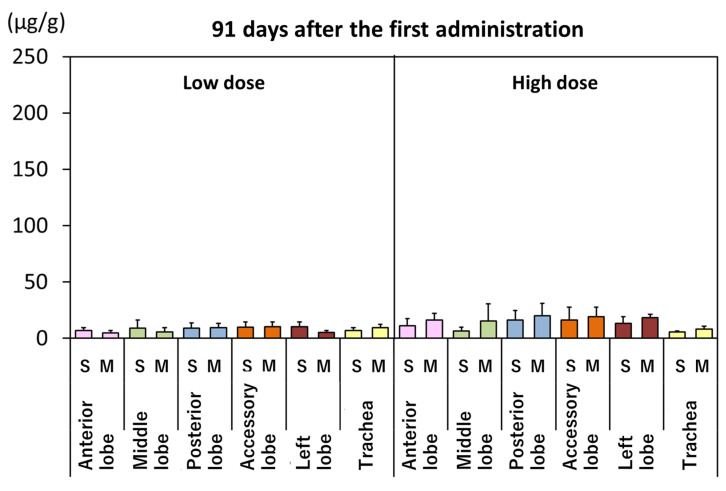
Distribution of MWCNT deposition in the lung (lung lobe) and trachea. S: single administration, M: four divided administrations (multiple administration).

The distribution of MWCNT deposition in each lung lobe and trachea is shown in Figure 9. MWCNT deposition is shown as the concentration per lung lobe weight (or trachea weight). MWCNT deposition in each lung lobe was higher in the posterior, accessory, and left lobes at 4 h after administration and at days 14 and 28 in both the low- and high-dose groups. However, there was almost no difference in MWCNT deposition at day 91. There were no obvious differences between the single- and four-administrations groups, and there were no obvious differences at any of the measurement time points. A certain amount of MWCNTs remained in the lungs and trachea in all the dosed groups in the low- and high-dose groups on day 91.

The clearance of MWCNTs from the lungs was over 50% at day 14 in all administration groups. The clearance rate of MWCNTs in the lungs during this period was determined to be 50% for the low-dose single-administration group, 52% for the low-dose four-administration group, 62% for the high-dose single-administration group, and 60% for the high-dose four-administration group. The half-life of MWCNT-7 in the lungs was calculated to be 14 days for the low-dose single-administration group, 16 days for the low-dose four-administration group, and 12 days for both the single- and four-administration high-dose groups.

## 4. Discussion

MWCNTs were administered to rats by a single intratracheal administration or the same final dose as the single administration but divided over four administrations. Low and high doses of single and four intratracheal administrations of MWCNTs were used to examine lung toxicity and lung deposition and distribution. The dose for intratracheal administration was set to a dose that took into account the amount of MWCNTs deposited in the lungs in our previous 13-week inhalation study [1], as described in Section 2.3.

The effects of the intratracheal administration of MWCNTs were seen in the BAL, lung weights, and macroscopic and histopathological examination of lungs and lung-related lymph nodes. In the BAL cytological and biochemical examinations, parameters indicative of inflammatory reactions and cytotoxic changes were increased in a dose-dependent manner at the low and high doses. A comparison of the number of administrations showed a greater effect with four administrations compared to the single administration for both the low and high doses, especially in the BAL examination. Since the BAL examination was performed 14 days after the start of administration, there was a 7-day difference in the time between the last administration and the BAL examination between the single- and the four-administrations groups; the stronger effect of the four administrations could be due to the shorter time between the last administration and the BAL examination. Total protein, LDH, and ALP were slightly higher in the vehicle control group after four administrations than after a single administration, but this was due to Tween 80, a non-ionic surfactant used as a dispersing agent.

Lung weights increased in both the low- and high-dose groups at days 14 and 28. At day 91, lung weights increased only in the high-dose groups. A histopathologic examination of the lung revealed increased alveolar macrophages, the deposition of MWCNT fibers (phagocytosed by alveolar macrophages and MWCNT fibers that were not phagocytosed), granulomatous changes, and the deposition of MWCNT fibers in the BALT and lung-related lymph nodes in the low- and high-dose single-administration and four-administration groups at all examination time points. Alveolar macrophages phagocytosing MWCNTs were found in all animals of all dose groups, higher grade at high doses on days 14 and 28, but lower grade on day 91. MWCNT deposition in the BALT and lung-related lymph nodes was seen in more rats on days 28 and 91 than on day 14. This indicated that the MWCNTs were removed from the lungs over time. Alveolar type II epithelial hyperplasia was observed on day 91, and fibrosis of the alveolar wall was seen in a small number of cases on days 28 and 91.

Although there was no difference in many histopathological findings when comparing a single administration and four administrations, MWCNT deposition in the BALT tended to be more common after a single administration. In contrast, deposition in the lung-related lymph nodes was more common in the four-administration groups, at both the low and high doses. The difference in the amount of MWCNT deposition in the BALT and lung-associated lymph nodes in the four-administration groups may be related to the amount of MWCNT solution administered into the lung per administration. It is thought that MWCNTs migrated from the lungs to the lung-associated lymph nodes when administered over the course of four administrations, because four times as much volume of solution (liquid) is administered compared to a single dose, and therefore, this increase in solution may aid MWCNT migration from the lungs to the lymph nodes.

MWCNTs are rapidly removed from the lungs by the mucociliary escalator and by phagocytosis and removal by alveolar macrophages. After this, they are slowly removed from the lung by the lymphatic flow [18]. With intratracheal administration, the slow removal from the lungs after day 14 to day 91 is probably due to removal by the lymphatic flow and the trapping of MWCNT fibers in lymph nodes. Comparing inhalation exposure and intratracheal administration, in inhalation exposure fibers gradually accumulate in the lungs through respiration, whereas in intratracheal administration large amounts of fibers and fiber suspension fluids are delivered into the lungs This suggests that intratracheal administration is likely to differ from inhalation exposure in the method by which the fibers are removed from the lung, with lymphatic flow having a more prominent role in removal of fibers from the lung after intratracheal administration.

Regarding the analysis of MWCNT deposition in the lung, there was no significant difference between a single and four administrations in either the low-dose or high-dose groups. MWCNT lung deposition decreased significantly on day 14 compared to hour 4, followed by a linear and gradual decrease from day 14 to days 28 and 91 (Figure 8).

In the study on the clearance of MWCNTs after inhalation exposure at the JBRC [17], the deposition was halved from immediately after exposure to the day after exposure, and thereafter the clearance rate was extremely low. Specifically, when the day after exposure was used as the base, the deposition fraction after 56 days was 88% of the deposition the day after exposure (a clearance rate of 12% per 56 days). On the other hand, in the present study based on the amount of deposition after 14 days, the deposition after 91 days was 42% in the high-dose four-administrations group (a 58% clearance from day 14 to day 91) and 60% in the low-dose single-administration group (a 40% clearance from day 14 to day 91). The excretion rate in the high-dose single-administration group was slightly higher than in the high-dose four-administrations group, and the excretion rate in the low-dose four-administrations group was slightly lower than that in the low-dose single-administration group; however, the excretion rates in the high-dose groups were nearly equivalent, and the excretion rates in the low-dose groups were nearly equivalent (Figure 8). Thus, intratracheal administration had a higher excretion rate than inhalation exposure.

An analysis of MWCNT deposition in each lung lobe showed a tendency for MWCNTs to be found more in the left lobe and in the posterior and accessory lobes of the right lung at days 14 and 28, but no clear differences were found at day 91. This trend was also observed in the histopathology of the lung, where MWCNTs tended to be found more in the posterior and accessory lobes of the right lung and more in the posterior side of the left lung. This difference in the distribution of MWCNTs in different lung lobes was not seen in our MWCNT inhalation exposure studies [1,17]. The different distribution of MWCNT deposition in the inhalation and intratracheal administration studies is considered to be due to the position of the animal during intratracheal administration. During intratracheal administration, the animal was held with the head in the up position. In this position, the MWCNTs were more likely to enter the posterior side of the left lung and the posterior lobe and accessory lobe of the right lung and were less likely to enter the right anterior lobe and the anterior side in left lung, which are located in the upper part of the lung. This tendency for a non-homogeneous distribution in the lungs did not change with the four divided administrations, and there was no tendency for the four divided administrations to enter the lungs more easily in the anterior direction. In previous nano-NiO administration experiments, it was also observed that the non-homogeneous distribution in the lungs did not become homogeneous after multiple administrations [4].

A comparison of the histopathological findings between the previous 13-week MWCNT inhalation exposure study and present study showed that the lung findings seen in the inhalation exposure study were also seen in the intratracheal administration study. However, alveolar type II epithelial hyperplasia was not seen after inhalation exposure but was seen in many animals after intratracheal administration. In addition, fibrosis of the alveolar wall occurred more frequently with inhalation exposure than with intratracheal administration. Thus, both intratracheal administration and inhalation exposure induced lung epithelial and stromal changes in rats, and the lesions were qualitatively similar. However, it has been suggested that intratracheal administration may induce more severe epithelial lesions in the lung. As stated above, these results allow us to conduct a long-term intratracheal administration study for MWCNT carcinogenicity. Morimoto et al. [6] reported a difference in pulmonary inflammation that was observed between the high- and low-toxicity nanomaterials in intratracheal instillation studies and in inhalation studies, suggesting that intratracheal instillation studies may be useful for ranking the harmful effects of nanoparticles. Osier and Oberdörster [7] also mentioned a difference in the pulmonary response to inhaled vs instilled titanium dioxide particles, which may be due to differences in dose rate, particle distribution, or clearance between the two methods. Based on our results and those shown above, we believe that intratracheal administration is a useful method for evaluating the toxicity of various nanomaterials, not just MWCNTs. In our previous nano-NiO intratracheal study using single and multiple intratracheal administrations, the response induced by nano-NiO was stronger after four administrations of the test substance [4]. Therefore, four administrations are better for confirming lung toxicity, but since both substances showed sufficient responses after a single administration, it may be considered to be less necessary to administer four administrations when evaluating toxicity. Multiple administrations may be useful when the test substance is highly viscous and administered at lower concentrations, or when the test substance can only be controlled at a lower concentration. Physical properties and toxicity information should be researched in advance, and experiments should be planned with an understanding of whether or not the substance is highly toxic.

In conclusion, lung toxicity due to the intratracheal administration of MWCNTs was compared after a single administration of MWCNTs or the same final dose as the single administration but divided over four administrations at low and high doses of MWCNTs. In the BAL examination, the parameters indicating an inflammatory response and cytotoxicity showed a stronger response after four administrations, and both were elevated in the low- and high-dose groups. The histopathologic changes showed increased alveolar macrophages, MWCNT deposition (phagocytosed by alveolar macrophages and MWCNT fibers that were not phagocytosed), granulomatous changes, and MWCNT deposition in the BALT and lung-related lymph nodes. In addition, alveolar type II epithelial hyperplasia and fibrosis of the alveolar wall were observed at later time points. MWCNTs are deposited in response to concentration in both single and multiple administrations. The results were almost the same whether the MWCNTs were administered once or four times. In addition, some lung lobes were difficult for MWCNTs to enter when administered intratracheally. We concluded that a toxicity evaluation can be sufficiently performed even with a single intratracheal administration.

## Figures and Tables

**Table 1:** Number of animals used for each examination and date of examination.

Group Name (Total Dose)	Examination	Date of Examination			
		4 h	Day 14	Day 28	Day 91
Untreated control group	BAL	-	5	-	-
Vehicle control group: Single	BAL	-	5	-	-
	Blood, Pathology	-	5	5	5
	Lung burden	-	-	-	-
MWCNTs group: Single (Low)	BAL	-	5	-	-
	Blood, Pathology	-	5	5	5
	Lung burden	5	5	5	5
MWCNTs group: Single (High)	BAL	-	5	-	-
	Blood, Pathology	-	5	5	5
	Lung burden	5	5	5	5
Vehicle control group: Multiple	BAL	-	5	-	-
	Blood, Pathology	-	5	5	5
	Lung burden	5	5	5	5
MWCNTs group: Multiple (Low)	BAL	-	5	-	-
	Blood, Pathology	-	5	5	5
	Lung burden	5	5	5	5
MWCNTs group: Multiple (High)	BAL	-	5	-	-
	Blood, Pathology	-	5	5	5
	Lung burden	5	5	5	5

BAL: bronchoalveolar lavage examination. Blood: blood sampling (hematological and blood biochemical analyses). Pathology: organ weights, macroscopic and histopathological examinations.

**Table 2:** Histopathological findings of the lung and lymph nodes (selected).

Day 14 after first administration				
Total dose (low: 240 μg/kg, high: 480 μg/kg)	Low		High	
**No. of times administered**	1	4	1	4
**No. of animals examined**	5	5	5	5
Lung				
increase in alveolar macrophage	+: 5	+: 5	++: 5	++: 5
deposit of MWCNTs (phagocytosed in alveolar space)	+: 5	+: 5	+: 5	+: 5
deposit of MWCNTs (non-phagocytosed in alveolar space)	+: 5	+: 5	+: 5	+: 5
alveolar type II epithelial hyperplasia	0	0	+: 1	0
granulomatous change	0	+: 1	+: 4	+: 2
fibrosis of alveolar wall	0	0	0	0
deposit of MWCNTs (in BALT)	0	0	+: 4	0
Lung-associated lymph nodes				
deposit of MWCNTs	0	0	0	+: 3
**Day 28 after first administration**				
**Total dose (low: 240 μg/kg, high: 480 μg/kg)**	**Low**		**High**	
**No. of times administered**	1	4	1	4
**No. of animals examined**	5	5	5	5
Lung				
increase in alveolar macrophage	+: 5	+: 5	++: 5	++: 5
deposit of MWCNTs (phagocytosed in alveolar space)	+: 5	+: 5	+: 5	+: 5
deposit of MWCNTs (non-phagocytosed in alveolar space)	+: 5	+: 5	+: 5	+: 5
alveolar type II epithelial hyperplasia	0	0	0	+: 1
granulomatous change	+: 1	0	+: 3	+: 2
fibrosis of alveolar wall	0	0	+: 1	0
deposit of MWCNTs (in BALT)	+: 4	+: 3	+: 5	+: 2
Lung-associated lymph nodes				
deposit of MWCNTs	0	0	0	+: 4
**Day 91 after first administration**				
**Total dose (low: 240 μg/kg, high: 480 μg/kg)**	**Low**		**High**	
**No. of times administered**	1	4	1	4
**No. of animals examined**	5	5	5	5
Lung				
increase in alveolar macrophage	+: 5	+: 5	+: 5	+: 5
deposit of MWCNTs (phagocytosed in alveolar space)	+: 5	+: 5	+: 5	+: 5
deposit of MWCNTs (non-phagocytosed in alveolar space)	+: 5	+: 5	+: 5	+: 5
alveolar type II epithelial hyperplasia	+: 3	+: 3	+: 5	+: 5
granulomatous change	+: 3	+: 1	+: 5	+: 5
fibrosis of alveolar wall	0	0	+: 1	+: 1
deposit of MWCNTs (in BALT)	+: 5	+: 2	+: 5	+: 4
Lung-associated lymph nodes				
deposit of MWCNTs	0	+: 4	+: 3	+: 5

+: slight, ++: moderate, BALT: bronchus-associated lymphoid tissue.

## Data Availability

The datasets used and analyzed during the current study are available from the corresponding authors on reasonable request.
